# Cytogenetic and molecular characteristics of *Potamotrygon motoro* and *Potamotrygon* sp. (Chondrichthyes, Myliobatiformes, Potamotrygonidae) from the Amazon basin: Implications for the taxonomy of the genus

**DOI:** 10.1590/1678-4685-GMB-2020-0083

**Published:** 2021-04-07

**Authors:** Vanessa Paes da Cruz, Maria Ligia Oliveira Nobile, Fabilene Gomes Paim, Aisni Mayumi Correia de Lima Adachi, Giovana da Silva Ribeiro, Daniela Carvalho Ferreira, José Carlos Pansonato-Alves, Patrícia Charvet, Claudio Oliveira, Fausto Foresti

**Affiliations:** 1 Universidade Estadual Paulista (UNESP), Instituto de Biociências de Botucatu, Laboratório de Biologia e Genética de Peixes, Botucatu, SP, Brazil.; 2 Universidade Federal de Mato Grosso (UFMT), Instituto de Biociências, Departamento de Biologia e Zoologia, Cuiabá, MT, Brazil.; 3 Universidade Federal do Paraná (UFPR), Departamento de Engenharia Ambiental, Laboratório de Ecologia e Conservação, Curitiba, PR, Brazil.; 4 Universidade Federal do Ceará (UFC), Departamento de Biologia, Programa de Pós-Graduação em Sistemática, Uso e Conservação da Biodiversidade, Fortaleza, CE, Brazil.

**Keywords:** Potamotrygon, freshwater stingrays, fish chromosomes, DNA barcode, biodiversity

## Abstract

The chromosomes of two freshwater stingrays, *Potamotrygon motoro* and *Potamotrygon* sp., from the Amazon River basin in Brazil were investigated using integrated molecular (cytochrome c oxidase subunit 1) and cytogenetic analyses. *Potamotrygon motoro* presented intraspecific variation in the diploid number, with 2n=66 in the females and 2n=65 in the males, while *Potamotrygon* sp. had a karyotype with 66 chromosomes, in both sexes. The C-banding revealed the presence of heterochromatic blocks accumulated in the centromeric region of all the chromosomes in both species. The FISH assays with 18S DNA probes highlighted the terminal region of three or four chromosome pairs in *P. motoro* and seven chromosomes in *Potamotrygon* sp. The rDNA 5S sequences were found in only one chromosomal pair in both species. The interspecific genetic distance based on the COI sequences, between *P. motoro* and *Potamotrygon* sp*.* from Amazon River was 10.8%, while that between the Amazonian *P. motoro* and *Potamotrygon amandae* from the Paraná River was 2.2%, and the genetic distance between *Potamotrygon* sp. and *P. amandae* was 11.8%. In addition to the new insights on the cytogenetics of the study species, the results of the present study confirmed the existence of heteromorphic sex-linked chromosomes in *P. motoro*.

The subfamily Potamotrygoninae is the only group of stingrays that radiated into freshwater environments (Lovejoy, 1996; Toffoli *et al*., 2008, Last *et al*., 2016). Four valid genera are currently recognized in this subfamily - *Paratrygon* Duméril, 1865, *Potamotrygon* Garman, 1877, *Plesiotrygon* Rosa, Castello and Thorson, 1987, and *Heliotrygon* Carvalho and Lovejoy, 2011 (Rosa *et al*., 1987; Carvalho *et al*., 2003, 2011). *Paratrygon* is monospecific, while *Heliotrygon* and *Plesiotrygon* each have two valid species (Carvalho *et al*., 2011; Carvalho *et al*., 2016b; Last *et al*., 2016). *Potamotrygon* is the most specious genus, with 31 taxa (Loboda and Carvalho, 2013; Carvalho *et al*., 2016a; Nelson *et al*., 2016; Fontenelle and Carvalho, 2017; Fricke *et al*., 2020).

The potamotrygonins are elasmobranchs that are fully adapted to freshwater environments, and are restricted to the continental waters of South America. In northern South America, potamotrygonins inhabit the hydrographic basins that drain into the Atlantic Ocean and the Caribbean Sea, while in southern South America, the are found in the Paraná-Paraguay river basin, which drains into the Atlantic Ocean (Thorson *et al*., 1983; Carvalho *et al*., 2003; Rosa *et al*., 2010). Ongoing research into the evolution of these fish has emphasized the need for a well-supported phylogeny that can provide systematic insights into the evolutionary processes that determined the characteristics of the potamotrygonins (Aschliman, 2011).

Potamotrygonins are targeted intensively by fisheries, including the ornamental fish trade, which has led to the classification of some species as vulnerable or even endangered, although the majority are listed as data deficient by the International Union for the Conservation of Nature (IUCN). This scenario is exacerbated by the biological characteristics of the elasmobranchs, including their reduced fecundity, slow growth, and late sexual maturation, together with a lack of adequate management planning and conservation measures (Charvet-Almeida *et al*., 2005; Duncan *et al*., 2010).

The cytogenetics of this group of organisms is characterized by the extensive variability in the chromosomal complement found among the species studied to date which may provide important insights into the mechanisms of the evolutionary diversification of this group (Rocco *et al*., 2005). Up to now, however, cytogenetic data are available for only a small number of the potamotrygonins species, but despite this, the variation in the chromosome number and the absence of a predominant karyotype formula for these fish is typical of both marine and freshwater stingrays (Stingo and Rocco, 1991; Rocco *et al*., 2005, 2006, 2007; Valentim *et al*., 2006, 2013, 2014, 2019; Cruz *et al*., 2011; Aichino *et al*., 2013).

The present study investigated the stingrays of the Amazon region, evaluating the applicability of chromosomal markers and DNA barcoding for the identification of *Potamotrygon motoro* and *Potamotrygon* sp., and to provide systematic insights for the chromosomal evolution and taxonomy of the species of this group.

The analyses presented here were conducted on specimens of *Potamotrygon motoro* and *Potamotrygon* sp. collected from the Amazon River (-2.789890/ -57.918168) near the city of Manaus (Table S1, Figure S1). Samples of *Potamotrygon amandae* from the Paraná River basin were also included in the molecular analyses for comparison, given that the specimens from this basin were considered to be *P.* aff. *motoro* prior to the review of Loboda and Carvalho (2013). All the samples were collected in strict accordance with the regulations of the Brazilian Federal Animal Ethics Committee (SISBIO 13843-1), and the analyses followed the International Guidelines for Animal Experiments, as authorized by CEEAA IBB/UNESP, protocol number 556. A small fragment of muscle tissue (< 1 cm^2^) was collected from each individual and preserved in 96% ethanol, before being deposited in the museum of the Laboratory of Fish Biology and Genetics at UNESP in Botucatu, São Paulo, Brazil.

The chromosomal preparations were obtained from spleen cell suspensions following the technique described by Cruz *et al*. (2015). The distribution of the constitutive heterochromatin was investigated by C-banding (Sumner, 1972).

The 5S and 18S rDNA were mapped by fluorescence *in situ* hybridization (double-FISH) accord to Pinkel *et al*. (1986). The probes were obtained by PCR (Polymerase Chain Reaction) from the total DNA of *P. motoro* using the primers 5SA (5’-TCAACCAACCACAAAGACATTGGCAC-3’) and 5SB (5’-TAGACTTCTGGGTGGCCAAAGGAATCA-3’) (Pendás *et al*., 1994), and NS18 (5’-GTAGTCATATGCTTGTCTC-3’) and NS18 (5’-TCCGCAGGTTCACCTACGGA-3’) (White *et al*., 1990). For *P. motoro*, the 18S rDNA probe was labeled with biotin-16-dUTP (Roche) and the 5S rDNA probe with digoxigenin-11-dUTP (Roche), whereas for *Potamotrygon* sp., the 18S rDNA probe was labeled with digoxigenin-11-dUTP and the 5S rDNA probe with biotin-16-dUTP. The signals were detected by fluorescein-conjugated avidin (FITC, Sigma-Aldrich) and anti-digoxygenin-rhodamine (Roche). The chromosomes were subsequently counterstained with 4,6-diamidino-2-phenylindole, or DAPI (Vector).

The metaphase spreads were analyzed and photographed using an Olympus BX61 photomicroscope with an attached a DP70 digital camera, using the Image ProPlus 6.0 software (Media Cybernetics, Rockville, Md, USA). The chromosomes were classified as metacentric (m), submetacentric (sm), subtelocentric (st), and acrocentric (a), following Levan *et al*. (1964).

For the molecular analysis, the genomic DNA was extracted from muscle tissue that had been preserved in 95% ethanol using a DNeasy Blood and Tissue kit (Qiagen, Hilden, Germany), following the manufacturer’s instructions. A partial sequence of the cytochrome c oxidase subunit I (COI) gene was used for the molecular identification of the potamotrygonins species. This sequence was obtained by PCR amplification using the FishF1 (5’-TCAACCAACCACAAAGACATTGGCAC-3’) and FishR1 (5’-TAGACTTCTGGGTGGCCAAAGAATCA-3’) primers described by Ward *et al*. (2005). The COI was amplified by PCR in a 12.5 μL reaction volume containing 1.25 μL of 10 × PCR buffer, 0.25 μL of MgCl2 (50 mM), 0.2 μL of dNTPs (2 mM), 0.5 μL of each primer (10 μM), 0.1 μL of 1.25 U *Taq* platinum DNA polymerase, and 1 μL of the DNA template (100 ng). The PCR protocol was 94 ºC for 5 min, followed by 30 cycles of 94 ºC for 40 s, 52 ºC for 30 s, and 72 ºC for 1 min, with a final extension at 72 ºC for 8 min. The PCR products were visualized in 1% agarose gel and purified by ExoSAP-IT (USB Europe GmbH, Staufen, Germany), incubated at 37 ºC for 60 min, and then at 80 ºC for 15 min. The samples were used as sequencing templates in an automatic ABI 3730 capillary sequencer using the BigDye Terminator v.3.1 Cycle Sequencing kit (Applied Biosystems, Inc.), following the manufacturer’s instructions, and were sequenced in an ABI 3130X1 Genetic Analyzer (Applied Biosystems).

The sequences were aligned using Geneious 4.8.5 (Drummond *et al*., 2009), and submitted to the GenBank. A Neighbor-joining (NJ) analysis was used to construct a tree of pairwise distances, which was estimated using the Kimura-2-parameter model, run in MEGA version 6 (Tamura *et al*., 2013), and tested by the bootstrap method, with 1000 pseudoreplicates (Felsenstein, 1985). The tree was visualized and edited using Figtree 1.4.2 software (Rambaut and Drummond AJ, 2014; http:/tree.bio.ed.ac.uk/software/figtree). The COI sequences of *histrix* (GenBank accession JN18407) and *Hypanus guttatus* (GenBank accession JX034000) were obtained from the GenBank and inserted together with the alignments for the construction of more robust dendrograms. Details of the samples and their GenBank accession numbers are provided in Table S1, which also shows the distribution of the collection sites.

All the male individuals of *P. motoro* had a karyotype of 65 chromosomes, with a karyotype formula of 20m + 9sm + 10st + 26a (Figure 1A), while a diploid number of 2n=66 chromosomes was observed in the females, with a formula of 20m + 10sm + 10st + 26a (Figure 1B). The C-banding in *P. motoro* revealed the presence of small heterochromatic blocks accumulated in the centromeric region of all the chromosomes of all the karyotype, in addition to a conspicuous block in the long arm of pair 14 (Figure 1A, B). The double-FISH with the 18S rDNA probe revealed eight positive signals, four in metacentric chromosomes and four in acrocentric chromosomes, while the 5S rDNA probe revealed two signals in the interstitial region of the long arm of the submetacentric chromosomes (Figure 2A).

**Figure 1 -:**
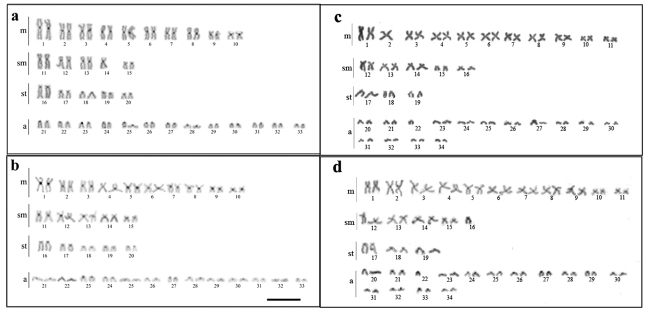
Karyotypes of (a) male and (b) female *Potamotrygon motoro* after C-banding. Karyotype of *Potamotrygon* sp. stained with (c) Giemsa and (d) after C-banding. Scale bar =10 µm.

**Figure 2 -:**
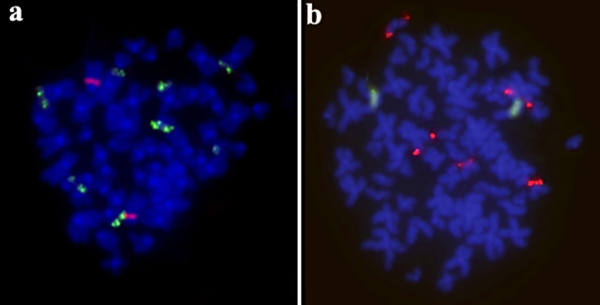
(a) Metaphase of *P. motoro* showing the 5S rDNA (red) and 18S (green) fluorescent signals; (b) Metaphase of *Potamotrygon* sp. showing the rDNA 5S (green) and 18S (red) fluorescent signals.

All the *Potamotrygon* sp. individuals analyzed had a karyotype with 2n=66 chromosomes, with the karyotype formula varying among individuals, which were either 21m + 10sm + 6st + 29a or 22m + 9sm + 6st + 29a (Figure 1C and 1D). This variation was due to presence of chromosomes without homologs in the metacentric pair 2 and acrocentric pair 22 (Figure 1C), whereas in other specimens, the homologs were absent in submetacentric pair 16 and pair 22 (Figure 1D). As both male and female individuals of *Potamotrygon* sp. were analyzed, a polymorphism related to the presence of sex chromosome cannot be ruled out, although further research will be needed to confirm this scenario. The C-banding revealed an accumulation of constitutive heterochromatin in the centromeric region of all the chromosomes (Figure 1C and 1D). The FISH with the 18S rDNA probes revealed positive signals in two metacentric chromosomes and three acrocentric chromosomes, while the 5S rDNA probes highlighted signals in one chromosome pair (Figure 2B).

The alignment of the COI sequence comprised 630 sites, of which, 80 were variable and 76 were informative for parsimony analysis. The mean nucleotide composition was 28% adenine (A), 29.4% cytosine (C), 16.4% guanine (G), and 26.3% thymine (T). The NJ tree had three well-supported lineages (Figure 3). The first lineage corresponds to the *P. amandae* samples from the Paraná basin, the second to the *P. motoro* samples from the Amazon basin, while the third included both the *Potamotrygon* sp. samples from the Amazon and the *P. histrix* sequences from “Brazil” (Aschliman, 2011). The smallest intraspecific distance value in the dataset was 0.1%, recorded in *P. amandae*, while the largest intraspecific distance (0.9%) was observed in *P. motoro*. Intermediate distances (mean = 0.3%) were recorded in the third lineage (*Potamotrygon* sp. *+ P. histrix*).

**Figure 3 -:**
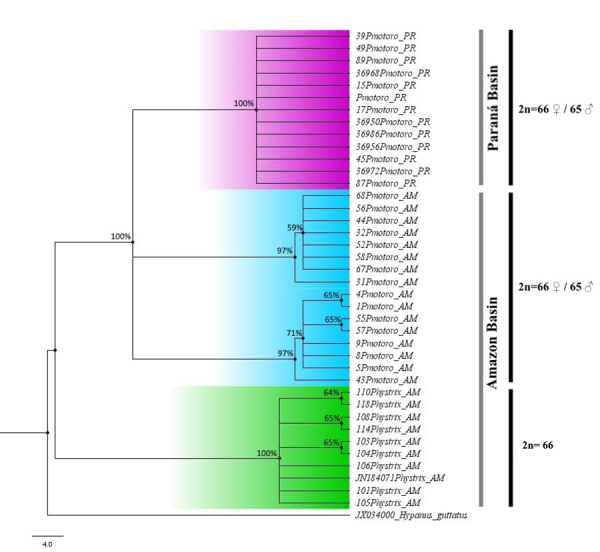
Neighbor-Joining tree of the COI gene sequence and the sex chromosome systems found in potamotrygonins species, including *Potamotrygon amandae* from the Paraná basin (purple), *P. motoro* (blue) and *Potamotrygon* sp. from the Amazon basin, and *P. histrix* (JN184071 *“P. hystrix”*) (green). The bootstrap values are shown at the branch nodes.

Despite the high degree of genetic similarity recorded between the *P. P. histrix* sequence of Aschliman (2011) and the *Potamotrygon* sp. sequence in the present study, the type locality of *P. histrix* is in the Paraná-Paraguay basin (Rosa, 1985; Nion *et al*., 2002; Carvalho *et al*., 2003; Araújo *et al*., 2004; Carvalho, 2016), and the species is known to occur in the Amazon basin. The voucher specimen (ZMB 16863) identified as *P. histrix* by Aschliman (2011) is deposited in the fish collection of the Museum für Naturkunde in Berlin, Germany, which impedes the confirmation of the identification of the specimen. This voucher specimen probably belongs to the group of freshwater stingrays that have a reticulated dorsal color pattern, which bear some resemblance to *P. histrix*.

The online data available on the capture location of this specimen refer only to Brazil, but provide no further details. Some of the specimens collected in the present study, in the principal Solimões-Amazon channel, downstream from Manaus, also belong to the reticulated group, but as no specimens were collected, it was impossible to confirm their identification, and the COI marker did not provide a clear differentiation. Given this, the specimens were identified here only as *Potamotrygon* sp., and as they were 99.6% similar to Aschliman (2011) *Potamotrygon histrix* (JN184071 “*P. hystrix*”), they may in fact correspond to *Potamotrygon orbignyi*, *Potamotrygon humerosa* or *Potamotrygon constellata*, given the region in which they were collected.

The interspecific genetic distance between *P. amandae* (Paraná basin) and *P. motoro* (Amazon basin) was 2.2%, while it was 10.8% between *P. motoro* and *Potamotrygon* sp.*/P. histrix*, and 11.8% between *P. amandae* and *Potamotrygon* sp.*/P. histrix*.

The potamotrygonins originated from a marine ancestor that invaded the freshwater environments of South America following the marine transgressions that occurred in the northwestern Amazon basin during the Miocene (Lovejoy, 1996; Carvalho *et al*., 2004). This ancestor subsequently dispersed widely, radiating in the freshwater environments of South America (Toffoli *et al*., 2008). Rocco *et al*. (2007) observed that marine rays have high diploid numbers (above 90 chromosomes) dominated by one-armed chromosomes and the presence of microchromosomes, as observed in *Raja asterias* (2n = 98). The diploid number of other species, such as *Myliobatis aquila* is lower, with a progressive increase in the number of bi-armed chromosomes. Rocco *et al*. (2007) concluded that Robertsonian rearrangements, primarily fusions, followed by inversions, were the principal mechanism of karyotype evolution in the stingrays (Rocco *et al*., 2007).

In the potamotrygonins, there is a reduction in the diploid number, from *Paratrygon aiereba*, which has 2n = 90 chromosomes (Table 1) and a large number of acrocentric chromosomes, to the specie of *Plesiotrygon iwamae* which has 2n=74 chromosomes, and species of the genus *Potamotrygon*, which have 2n = 65-68 chromosomes (Table 1). As there is a reduction in the number of acrocentric chromosomes, chromosomal rearrangements also certainly played an important role in the chromosomal evolution of these species.

**Table 1 -:** Summary of karyotypes information for the freshwater stingrays. 2n = diploid number.

Species	Collection sites		2n	Sexual system	References
	Country/Hydrographic basins	River			
*Paratrygon aiereba*	Brazil/ *Amazon basin*	middle Negro River	90	-	Valentim *et al*., 2006
*Plesiotrygon iwamae*	Brazil/ *Amazon basin*	middle Negro River	74	-	Valentim *et al*., 2019
*Potamotrygon amazona*	Brazil/ *Amazon basin*	middle Negro River	66♂	XY (?)	Valentim *et al*., 2019
*Potamotrygon constellata*	Brazil/ *Amazon basin*	Solimões River	66♀	-	Valentim *et al*., 2019
*Potamotrygon falkneri*	Brazil/ *Paraná basin*	upper Paraná River	66♀ / 65♂	X^1^X^1^X^2^X^2^/X^1^X^2^Y	Cruz *et al*., 2011
*Potamotrygon leopoldi*	Brazil/ *Amazon basin*	Xingu River	64	-	Valentim *et al*., 2019
*Potamotrygon motoro*	Brazil/ *Amazon basin*	middle Negro River	66	-	Valentim *et al*., 2006
	Brazil/ *Amazon basin*	Amazonas River	66 ♀ / 65 ♂	XX/X0	This study
*Potamotrygon amandae*	Argentina/ *Paraná basin*	Paraná River	66 ♀ / 65 ♂	X^1^X^1^X^2^X^2^/X^1^X^2^Y	Aichino *et al*., 2013
*Potamotrygon amandae*	Brazil/ *Paraná basin*	upper Paraná River	66♀ / 65♂	X^1^X^1^X^2^X^2^/X^1^X^2^Y	Cruz *et al*., 2011
*Potamotrygon sp.*	Brazil/ *Amazon basin*	Amazonas River	66	-	This study
*Potamotrygon orbignyi*	Brazil/ *Amazon basin*	Xingu River	66	XX/XY	Valentim *et al*., 2019
*Potamotrygon scobina*	Brazil/ *Amazon basin*	Solimões River	66♂	XY (?)	Valentim *et al*., 2019
*Potamotrygon wallacei*	Brazil/ *Amazon basin*	middle Negro River	67♀ / 68♂	XX/X0	Valentim *et al*., 2013
*Potamotrygon* aff. *wallacei*	Brazil/ *Amazon basin*	middle Negro River	68♀	-	Valentim *et al*., 2019

One other fundamentally important aspect of fish cytogenetics is the presence of sex chromosomes that have evolved through different mechanisms in the males and females. Sex-linked chromosomal heteromorphism linked to sex among stingrays have been described in *P. amandae*, *P*. *falkneri*, *P. motoro* (in present study), *P. amazona*, *P. orbignyi, P. scobina* and *P. wallacei* (Cruz *et al*., 2011; Aichino *et al*., 2013; Valentim *et al*., 2019) from the Paraná and Amazon basin, few species of *Potamotrygon* from the Amazon basin have been described with no sex chromosome system, among them *P. leopoldi, P. constellata, P. motoro* (Amazon basin) and *P.* aff. *wallacei* (Valentim *et al*., 2006, 2019).

Valentim *et al*. (2013) described the XX/X0 sex chromosome system in specimens of *Potamotrygon wallacei*, and this was considered to be a derived condition in the rays. However, the analysis of *Plesiotrygon iwamae*, a sister species of *Potamotrygon* (Carvalho *et al*., 2003; Carvalho and Lovejoy, 2011), did not reveal differentiated sex chromosomes (Valentim *et al*., 2013). On the other hand, the X1X1X2X2/X1X2Y system has been detected in *P. amandae* and *P. falkneri* from the upper Paraná basin (Cruz *et al*., 2011) and in *P. motoro* from the lower Paraná basin, in Argentina (Aichino *et al*., 2013). Both these systems were confirmed by the analysis of meiotic cells, and the different simple and multiple sex chromosome systems were both considered to represent derived traits in the chromosomal evolution process.

However, this type of heteromorphism was only found in these three species, and no sex-linked variation in chromosome number or morphology has been observed in other *Potamotrygon* species (Valetim *et al*., 2006, 2013). Even so, the full extent of the sex-linked chromosome systems of freshwater rays is probably underestimated, given the overall lack of cytogenetic data for this group.

The C-banding in *P. motoro* and *Potamotrygon* sp. revealed a similar distribution of constitutive heterochromatin to that found in other potamotrygonins, with conspicuous heterochromatic blocks being found primarily in the centromeric regions of the chromosomes of both freshwater and marine rays (Rocco *et al*., 2002, 2006; Valentim *et al*., 2006, 2013; Cruz *et al*., 2011). Overall, it would seem that the chromosome composition of the different rays of the superorder Batoidea are broadly similar.

Repetitive sequences have been mapped in a number of different marine rays (Rocco *et al*., 2002, 2005, 2007). In *Taeniura lymma* and *Raja montagui*, sequences of 5S rDNA were detected in two acrocentric chromosome pairs (Rocco *et al*., 2006). The present study is the first to provide data on the 5S and 18S rDNA sequences in freshwater stingrays. The 18S rDNA sequences were detected in a number of different chromosome pairs, representing a similar pattern to that found in the marine species (Rocco *et al*., 2006), while the 5S sequences were detected in only one of the chromosome pairs in each of the two species analyzed. It seems likely that the reduced distribution of the 5S rDNA sites in the *Potamotrygon* genome is the result of the chromosomal rearrangements that have occurred during the evolution of the superorder Batoidea.

The interspecific values of genetic distances recorded detected between the *P. motoro* samples from the Amazon basin and those of *P. amandae* from the Paraná basin, support the classification of these populations as distinct species, which reflect the differentiation of the *Potamotrygon* populations over their evolutionary history, as observed by Toffoli *et al*. (2008). Despite representing the same genus, *Potamotrygon* sp. was genetically distant from both *P. motoro* and *P. amandae*. Toffoli *et al*. (2008) recorded genetic distances between species of the genus *Potamotrygon* ranging from 1.9% between *P. orbignyi* and *P. scobina* to a maximum of 9.8% between *P. falkneri* and *Potamotrygon schroederi*.

Potamotrygonins have an ample variety of karyotypic formulae due to the chromosomal rearrangements that have occurred during the diversification of this group, in addition to a diversity of simple or multiple sex chromosome systems. These unique features are considered to be derived characters in the chromosomal evolution, and have only been found in the genus *Potamotrygon*, including *P. motoro* and *P. amandae* (Cruz *et al*., 2011; Aichino *et al*., 2013; Valentim *et al*., 2013). The combination of chromosomal and molecular analyses adopted in the present study revealed the complex characteristics of this stingrays and the possible existence of sibling or cryptic species.

## Supplementary material

The following online material is available for this article:

Table S1 - Species, locality, sequence ID, sex, analyses and the GenBank access of specimens of potamotrygonins (*P. motoro, P. amandae, Potamotrygon* sp., *P. histrix*) and *Hypanus guttatus.*

Figure S1 - South America/Brazil showing the sampling location of the potamotrygonins species (black star) in the Amazon basin.
