# Erratum

**DOI:** 10.1590/1678-4685-GMB-2020-0083er

**Published:** 2021-05-03

In the article “Cytogenetic and molecular characteristics of *Potamotrygon motoro* and *Potamotrygon* sp. (Chondrichthyes, Myliobatiformes, Potamotrygonidae) from the Amazon basin: Implications for the taxonomy of the genus”, with DOI number 10.1590/1678-4685-GMB-2020-0083, published in the journal *Genetics and Molecular Biology* issue 44(2):e20200083, **on page 1** the **author** name spelled as Daniela Carvalho Ferreira **should read** Daniela Cristina Ferreira.

