# Supplementary material for: Cytogenetic and molecular characteristics of Potamotrygon motoro and Potamotrygon sp. (Chondrichthyes, Myliobatiformes, Potamotrygonidae) from the Amazon basin: Implications for the taxonomy of the genus
Source: Genet Mol Biol. 2021 Apr 7;44(2):e20200083. doi: 10.1590/1678-4685-GMB-2020-0083 (PMC8033572; doi:10.1590/1678-4685-GMB-2020-0083)
Supplement: Table S1 - [file 1415-4757-GMB-44-2-e20200083-s1.pdf]

*Genetics and Molecular Biology*
https://doi.org/10.1590/1678-4685-GMB-2020-0083

# Supplementary Material to “Cytogenetic and molecular characteristics of Potamotrygon motoro and Potamotrygon sp. (Chondrichthyes, Myliobatiformes, Potamotrygonidae) from the Amazon basin: Implications for the taxonomy of the genus”

**Table S1.** Species, locality, sequence ID, sex, analyses and the GenBank access of specimens of potamotrygonins (*P. motoro*, *P. amandae*, *Potamotrygon* sp., *P. histrix*) and *Hypanus guttatus.*

| Species | Locality | Sequence ID | Sex | Analyses | Genbank access | Reference |
|---|---|---|---|---|---|---|
| *Potamotrygon amandae* | Paraná River basin | 39 P. amandae | Female | Molecular/COI | MK520997 | this study |
| *Potamotrygon amandae* | Paraná River basin | 49 P. amandae | Female | Molecular/COI | MK520998 | this study |
| *Potamotrygon amandae* | Paraná River basin | 89 P. amandae | Male | Molecular/COI | MK521001 | this study |
| *Potamotrygon amandae* | Paraná River basin | 36968 P. amandae | Male | Molecular/COI | MK521007 | this study |
| *Potamotrygon amandae* | Paraná River basin | 15 P. amandae | Female | Molecular/COI | MK520999 | this study |
| *Potamotrygon amandae* | Paraná River basin | 36980 P. amandae | Female | Molecular/COI | MK521003 | this study |
| *Potamotrygon amandae* | Paraná River basin | 17 P. amandae | Female | Molecular/COI | MK521002 | this study |
| *Potamotrygon amandae* | Paraná River basin | 36950 P. amandae | Female | Molecular/COI | MK521009 | this study |
| *Potamotrygon amandae* | Paraná River basin | 36986 P. amandae | Female | Molecular/COI | MK521005 | this study |
| *Potamotrygon amandae* | Paraná River basin | 36956 P. amandae | Female | Molecular/COI | MK521008 | this study |
| *Potamotrygon amandae* | Paraná River basin | 45 P. amandae | Female | Molecular/COI | MK521004 | this study |
| *Potamotrygon amandae* | Paraná River basin | 36972 P. amandae | Female | Molecular/COI | MK521006 | this study |
| *Potamotrygon amandae* | Paraná River basin | 87 P. amandae | Male | Molecular/COI | MK521000 | this study |
| *Potamotrygon motoro* | Amazon River basin | 68 P. motoro | Male | Molecular/COI | MK521020 | this study |
| *Potamotrygon motoro* | Amazon River basin | 56 P. motoro | Female | Cytogenetic + molecular/COI | MK521022 | this study |
| *Potamotrygon motoro* | Amazon River basin | 44 P. motoro | Female | Cytogenetic + molecular/COI | MK521024 | this study |
| *Potamotrygon motoro* | Amazon River basin | 32 P. motoro | Male | Cytogenetic + molecular/COI | MK521023 | this study |
| *Potamotrygon motoro* | Amazon River basin | 52 P. motoro | Female | Cytogenetic + molecular/COI | MK521021 | this study |
| *Potamotrygon motoro* | Amazon River basin | 58 P. motoro | Female | Cytogenetic + molecular/COI | MK521019 | this study |
| *Potamotrygon motoro* | Amazon River basin | 67 P. motoro | Female | Cytogenetic + molecular/COI | MK521025 | this study |

| Species | Locality | Sequence ID | Sex | Analyses | Genbank access | Reference |
|---|---|---|---|---|---|---|
| *Potamotrygon motoro* | Amazon River basin | 31 P. motoro | Male | Cytogenetic + molecular/COI | MK521018 | this study |
| *Potamotrygon motoro* | Amazon River basin | 4 P. motoro | Female | Cytogenetic + molecular/COI | MK521010 | this study |
| *Potamotrygon motoro* | Amazon River basin | 1 P. motoro | Male | Cytogenetic + molecular/COI | MK521011 | this study |
| *Potamotrygon motoro* | Amazon River basin | 55 P. motoro | Female | Cytogenetic + molecular/COI | MK521013 | this study |
| *Potamotrygon motoro* | Amazon River basin | 57 P. motoro | Male | Cytogenetic + molecular/COI | MK521014 | this study |
| *Potamotrygon motoro* | Amazon River basin | 9 P. motoro | Female | Molecular/COI | MK521017 | this study |
| *Potamotrygon motoro* | Amazon River basin | 8 P. motoro | Female | Molecular/COI | MK521016 | this study |
| *Potamotrygon motoro* | Amazon River basin | 5 P. motoro | Female | Molecular/COI | MK521015 | this study |
| *Potamotrygon motoro* | Amazon River basin | 43 P. motoro | Female | Molecular/COI | MK521012 | this study |
| *Potamotrygon sp.* | Amazon River basin | 110 Potamotrygon sp | Female | Cytogenetic + molecular/COI | MK520994 | this study |
| *Potamotrygon sp.* | Amazon River basin | 118 Potamotrygon sp | Female | Cytogenetic + molecular/COI | MK520995 | this study |
| *Potamotrygon sp.* | Amazon River basin | 108 Potamotrygon sp | Female | Cytogenetic + molecular/COI | MK520992 | this study |
| *Potamotrygon sp.* | Amazon River basin | 114 Potamotrygon sp | Female | Cytogenetic + molecular/COI | MK520993 | this study |
| *Potamotrygon sp.* | Amazon River basin | 103 Potamotrygon sp | Male | Cytogenetic + molecular/COI | MK520988 | this study |
| *Potamotrygon sp.* | Amazon River basin | 104 Potamotrygon sp | Male | Cytogenetic + molecular/COI | MK520989 | this study |
| *Potamotrygon sp.* | Amazon River basin | 106 Potamotrygon sp | Female | Molecular/COI | MK520996 | this study |
| *Potamotrygon sp.* | Amazon River basin | 101 Potamotrygon sp | Female | Molecular/COI | MK520991 | this study |
| *Potamotrygon sp.* | Amazon River basin | 105 Potamotrygon sp | Male | Molecular/COI | MK520990 | this study |
| *Potamotrygon histrix* | Brazil | JN184071 "P. hystrix" | no information | Molecular/COI | JN18407 | Aschliman 2011 |
| *Hypanus guttatus* | Atlantic Ocean | JX034000 *Hypanus guttatus* | no information | Molecular/COI | JX034000 | Ribeiro et al., 2012 |
