# Supplementary material for: Cytogenetic and molecular characteristics of Potamotrygon motoro and Potamotrygon sp. (Chondrichthyes, Myliobatiformes, Potamotrygonidae) from the Amazon basin: Implications for the taxonomy of the genus
Source: Genet Mol Biol. 2021 Apr 7;44(2):e20200083. doi: 10.1590/1678-4685-GMB-2020-0083 (PMC8033572; doi:10.1590/1678-4685-GMB-2020-0083)

Genetics and Molecular Biology
https://doi.org/10.1590/1678-4685-GMB-2020-0083

Supplementary Material to “Cytogenetic and molecular characteristics of Potamotrygon motoro and Potamotrygon sp. (Chondrichthyes, Myliobatiformes, Potamotrygonidae) from the Amazon basin: Implications for the taxonomy of the genus”

Figure S1. South America/Brazil showing the sampling location of the potamotrygonins species (black star) in the Amazon basin.

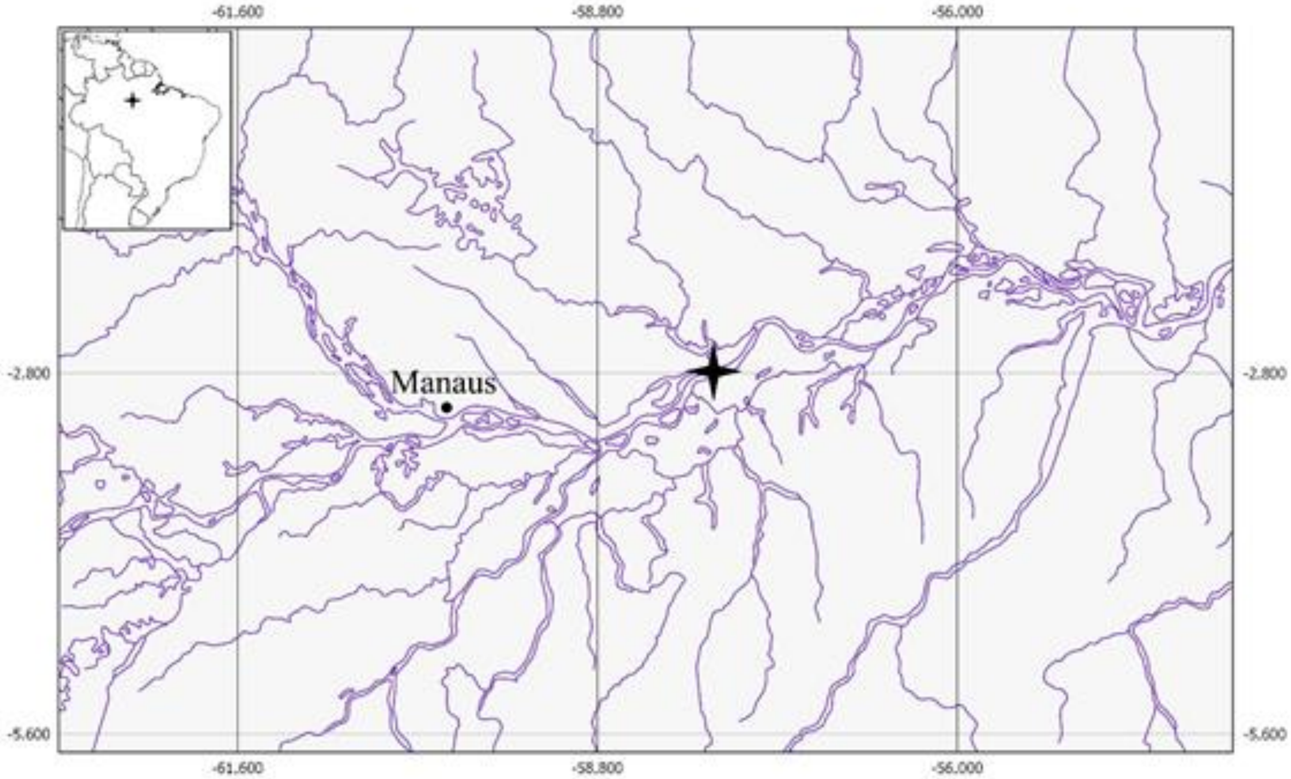

Supplement: Figure S1 - [file 1415-4757-GMB-44-2-e20200083-s2.pdf]
